# Conducting a secondary data analysis to estimate the incidence of congenital syphilis in South Africa, 2010 - 2016

**DOI:** 10.11604/pamj.supp.2018.30.1.15272

**Published:** 2018-05-18

**Authors:** Lazarus Rugare Kuonza, Tuyakula Nakale, Sinesia Lucinda Jose Sitao, Casey Daniel Hall

**Affiliations:** 1 South African Field Epidemiology Training Programme, National Institute for Communicable Diseases, National Health Laboratory Services, Johannesburg, South Africa; 2 School of Public Health, University of Witwatersrand, Johannesburg, South Africa; 3 School of Health Systems and Public Health, University of Pretoria, South Africa; 4 Namibia Institute of Pathology, Windhoek, Namibia; 5 Mozambican Field Epidemiology and Laboratory Training Programme; 6 Rollins School of Public Health, Emory University, Atlanta, USA

**Keywords:** Data management, data analysis, secondary data analysis, congenital syphilis, South Africa

## Abstract

Analysis of existing health data is often used as a cost-effective and time-efficient means to provide evidence to inform important public health decisions. However, the accuracy of the resultant decisions largely depends on the quality of the accessible data, and how the data are processed, analyzed, interpreted and reported. This case study, based on an actual secondary data analysis that was conducted by a trainee of the South African Field Epidemiology Training Programme during April 2017, was designed to provide a classroom simulation of practical considerations that should be taken into account when planning an analysis of a secondary dataset. The case study is ideally suited to reinforce principles already covered in lectures or in background reading assignments.

## How to use this Study

**General instructions:** the case study is suited for a class of 15-20 trainees per session. Trainees should preferably be seated in a U-shaped setup to encourage participation and interaction. The instructor facilitating the session should direct participants to read a paragraph aloud, going around the room to give each participant a chance to read. When a participant reads a question, the instructor may choose to engage the class in large group discussion of the answer, randomly identify a participant to respond to the question, or divide the class into smaller groups for exercises, depending on the type of question. The role of the instructor is largely to coordinate the session such that participants learn from each other, and not just from the instructor.

**Audience:** public health practitioners involved in the analysis and interpretation of surveillance data, and others who are interested in the topic.

**Prerequisites:** before using this case study, case study participants should have received lectures or other instruction on data quality, basic descriptive epidemiology, and analysis of secondary datasets.

**Materials needed:** flip chart or white board with markers, graph paper, and calculator.

**Level of training and associated public health activity:** data management and analysis.

**Time required:** 3 hours

**Language:** English

## Competing interest

The authors declare no competing interest.
